# Phenolics Profile, Antioxidant Activity and Flavor Volatiles of Pear Juice: Influence of Lactic Acid Fermentation Using Three *Lactobacillus* Strains in Monoculture and Binary Mixture

**DOI:** 10.3390/foods11010011

**Published:** 2021-12-21

**Authors:** Leyu Wang, Hexin Zhang, Hongjie Lei

**Affiliations:** College of Food Science and Engineering, Northwest A&F University, Xianyang 712100, China; wangleyu@nwafu.edu.cn (L.W.); zhanghexin1217@nwafu.edu.cn (H.Z.)

**Keywords:** pear juice, *Lactobacillus*, phenolics profile, antioxidant activity, flavor volatiles

## Abstract

The aim of this study was to evaluate the effects of lactic acid fermentation using three *Lactobacillus* strains (*Lactiplantibacillus plantarum* 90, *Lactobacillus helveticus* 76, *and Lacticaseibacillus casei* 37) in monoculture and binary mixture on phenolics profile, antioxidant activity and flavor volatiles in pear juice. Results showed that the colony counts of binary mixture were higher than monoculture in fermented pear juice. The total content of phenols was increased, while that of flavonoids was decreased significantly during fermentation (*p* < 0.05). Antioxidant activities in fermented peer juice including DPPH and ABTS radical scavenging abilities and ferric reducing antioxidant power (FRAP) were significantly improved (*p* < 0.05). Binary mixture of *Lactiplantibacillus plantarum* 90 and *Lacticaseibacillus casei* 37 fermentation exhibited strong DPPH radical scavenging ability, due to the increase in vanillic acid and arbutin contents. Furthermore, lactic acid fermentation improved the formation of alcohols, esters, acids and terpenoids, and reduced the contents of aldehydes and ketones. Thirty new compounds including 15 alcohols, seven esters, five acids, and three terpenoids were observed in fermented pear juice. Hierarchical cluster revealed that flavor volatiles in pear juice were improved dramatically by *Lactobacillus* strains fermentation, and there were dramatic differences between monoculture and binary mixture.

## 1. Introduction

Huangguan pear (*Pyrus bretschneideri* Rehd.), belonging to the *Rosaceae* family, is commonly consumed as fresh fruit, candy, preserved fruit, and syrup. Pear has traditionally been used as an important ingredient for treating ailments such as cough, constipation, diabetes, cardiovascular disease and alcoholism because of its antitussive, anti-inflammatory, anti-hyperglycemic and diuretic functions [1,2]. The major phenolic substances in pear are arbutin, caffeic acid, catechin, chlorogenic acid, epicatechin, ferulic acid, gallic acid, p-coumaric acid and rutin [3], which can regulate glucose and lipid metabolism in the human body [4].

There has been a significant increase in the demand for probiotic functional foods in recent years because of their nutrition and health benefits [5]. Non-dairy based probiotic functional foods development is booming because of vegetarianism, lactose intolerance, and high cholesterol risks [6]. Fruits and vegetables are rich in nutrients, such as vitamins, dietary fiber, carotenoids, folate, etc., and are considered as promising vehicles of probiotic microorganisms [7]. It has been demonstrated that lactic acid bacteria (LAB) can consume sugars as carbon sources to produce different metabolites, such as lactic acid, anti-hypertensive peptides, and exopolysaccharides [6,8,9]. Phenolics have been suggested to enhance the nutrition and sensory properties of fruit and vegetable products, and convert polyphenols to phenolic acids with lower molecular weight, which become more bioactive [8,10,11].

Although there are more and more studies related to LAB fermentation using various plant origin foods [1,12], information on changes in phenolics profile, antioxidant activity and flavor volatiles in pear juice after LAB fermentation is still scarce. Therefore, the present study aimed to evaluate the influence of three *Lactobacillus* strains (*Lactiplantibacillus plantarum* 90, *Lactobacillus helveticus* 76 and *Lacticaseibacillus casei* 37) in monoculture and binary mixture on polyphenols biotransformation, antioxidant activity and flavor volatiles in pear juice. The relationship between antioxidant activities and phenolic compounds was also investigated.

## 2. Materials and Methods

### 2.1. Preparation of Bacterial Strains

*Lactiplantibacillus plantarum* 90 (Lp90), *Lactobacillus helveticus* 76 (Lh76), and *Lacticaseibacillus casei* 37 (Lc37) were purchased from WECAREBIO company (Suzhou, China). All the LAB strains were stored as frozen stocks at −80 °C; in MRS broth with 50% glycerol (*w*/*v*). Glycerol stock (200 μL) was transferred into 40 mL of MRS broth, and cultivated at 37 °C for 12 h. Then, the above culture medium was sub-cultured in 100 mL of MRS broth and cultivated at 37 °C for 24 h. Cultures were collected by centrifugation (8000 rpm, 10 min, 4 °C) and cleaned with saline.

### 2.2. Preparation of Pear Juice and Fermentation

Huangguan pears (*Pyrus bretschneideri* Rehd.) were cleaned by potable water and squeezed by juicer (Midea, Foshan, China), then filtered to obtain pear juice. Pear juice was pasteurized immediately at 80 °C for 5 min. For fermentation, 0.5% (*v*/*v*) of monoculture or binary mixture strains at the ratio of 1:1 (*v*/*v*) were inoculated into pasteurized pear juice. Fermentation was carried out at 37 °C for 48 h. The pasteurized pear juice without inoculation was set as control.

### 2.3. Determination of Colony Counts

Colony counts were detected following the standard plate count method and diluted serially by sterile saline. Then, 100 μL aliquots of each dilution were inoculated separately onto MRS agar to conduct aerobic plate counts. The plates were inoculated at 37 °C for 48 h.

### 2.4. Determination of Physicochemical Properties

The pH, total soluble solids (TSS), titratable acid, total sugar and color were measured at intervals of 12 h. The pH was determined by pH meter (Mettler-Toledo, Greifensee, Switzerland). TSS (°Brix) was detected by digital refractometer (Atago, Tokyo, Japan). Titratable acid was detected through titration with 0.01 mmol/L NaOH and expressed as percent of lactic acid. Total sugar concentration was determined as glucose equivalents [13]. CIE color parameters were determined by Color Quest XE (HunterLab, Reston, VA, USA). Total color differences (Δ*E*) were calculated as follows:

(1) $$\Delta E=\sqrt{{\left(L_{0}-L\right)}^{2}+{\left(a_{0}-a\right)}^{2}+{\left(b_{0}-b\right)}^{2}}$$

### 2.5. Determination of Phytochemical Concentration

#### 2.5.1. Total Phenolic Content (TPC)

TPC was determined by the Folin-Ciocalteu method [14]. In brief, 0.5 mL of diluted pear juice was mixed with 2.5 mL of 10% Folin-Ciocalteu reagent. Then, 2 mL of 7.5% (*w*/*v*) Na_2_CO_3_ solution was added after 3 min. The mixture was subsequently incubated in the dark for 60 min, and the absorbance was measured at 765 nm with a UV-VIS spectrophotometer (Shimadzu, Kyoto, Japan).

#### 2.5.2. Total Flavonoid Content (TFC)

TFC was determined by aluminum chloride colorimetric method [15]. In brief, 0.5 mL of 50 g/L NaNO_2_ was added to 4 mL of pear juice and incubated for 5 min. Then, 1 mL of 100 g/L AlCl_3_ solution was added to the above mixture and incubated for 5 min. Subsequently, 2 mL of 2 mol/L NaOH was added and incubated for 10 min. The absorbance was read at 510 nm.

### 2.6. Determination of Antioxidant Activities

#### 2.6.1. DPPH Radical Scavenging Ability (RSA)

DPPH RSA was measured according to the previously reported method [16]. Diluted pear juice (2 mL) was added to 4 mL of DPPH methanol solution (45 mg/L). The mixture was shaken and left for 30 min in the dark, and then the absorbance (A) was read at 517 nm. Results were calculated as follows:

(2) $$DPPH\;RSA\;(\%)=\frac{A_{control}-A_{sample}}{A_{control}}\times 100$$

#### 2.6.2. ABTS Radical Scavenging Ability Assay (RSA)

ABTS RSA was measured according to the previously reported method [17]. In brief, 600 μL of diluted pear juice was added to 5.4 mL of ABTS working solution and incubated in the dark for 6 min. Then, absorbance was read at 734 nm. Results were calculated as follows:

(3) $$ABTS\;RSA\;(\%)=\frac{A_{control}-A_{sample}}{A_{control}}\times 100$$

#### 2.6.3. Ferric Reducing Antioxidant Power (FRAP) Assay

FRAP was detected according to the previously reported method [18]. In brief, 200 μL of diluted pear juice was added to 6 mL of FRAP working solution and incubated at 37 °C for 30 min. The absorbance was read at 593 nm.

### 2.7. Determination of Organic Acids and Phenolics Profile

Organic acids and phenolics profile were determined by HPLC (Shimadzu, Kyoto, Japan). Compounds were separated on a reverse-phase C18 column (4.6 mm × 250 mm, 5 μm, Waters, Milford, CT, USA). Organic acid contents were measured using UV detector at 210 nm [19]. The mobile phase was 0.01 mol/L KH_2_PO_4_-H_3_PO_4_ (pH 2.7) (solvent A) and methyl alcohol (solvent B). The proportion of solvent A to B was 97:3, and the flow rate was 0.6 mL/min. Phenolics profile was determined using UV detector at 280 nm [14]. Solvent A (0.1% formic acid) and solvent B (100% acetonitrile) were set as the following gradients: 12% B (25 min), 30% B (40 min), 45% B (50 min). After 60 min, solvent B dropped to 2%. Flow rate was 1.0 mL/min. Pear juices were filtered through a 0.45 μm membrane filter, and the injection volume was 10 μL. The concentrations of organic acids and phenolics profile were quantified by corresponding external standards (Sigma, St. Louis, MO, USA).

### 2.8. Determination of Flavor Volatiles

The flavor volatiles of pear juices were determined by GC-MS (Thermo, Waltham, MA, USA) [12]. Pear juice (5 mL) and 2 g of NaCl were added to a glass vial (15 mL). GC-MS equipped with a TR-5MS column (60 m × 0.25 mm, 0.25 μm, J&W Scientific, Palo Alto, CA, USA) and a quadrupole DSQ Ⅱ MS. Helium was the carrier gas and the flow rate was 1 mL/min. The semi-quantification of individual components was calculated based on 2-octanol as the internal standard (Sigma, USA).

### 2.9. Sensory Analysis

Sensory analysis of pear juices before and after LAB fermentation was carried out using a nine-point hedonic scale [20]. A 15-member semi-trained panel (eight women and seven men) was employed to evaluate the juices based on five sensory attributes (color, aroma, sweetness, sourness, and overall acceptability).

### 2.10. Statistical Analysis

All the experiments were performed in triplicate and results were presented as mean ± standard deviation (SD). Correlation analysis between phenolics profile and antioxidant activities was performed using R (ver. 3.2.2; The R Foundation for Statistical Computing, Vienna, Austria). The cluster heat map of volatiles was drawn using TBtools software (versions v1.098) with two-way hierarchical cluster dendrograms. SPSS (Version 26, IBM, Armonk, NY, USA) was used for ANOVA to compare the different results obtained from unfermented and fermented pear juices by monoculture and binary mixture of lactic acid bacteria. Tukey test was used to compute significant differences at *p* ˂ 0.05.

## 3. Results and Discussion

### 3.1. Colony Counts of LABs in Pear Juice during Fermentation

**Figure 1:**
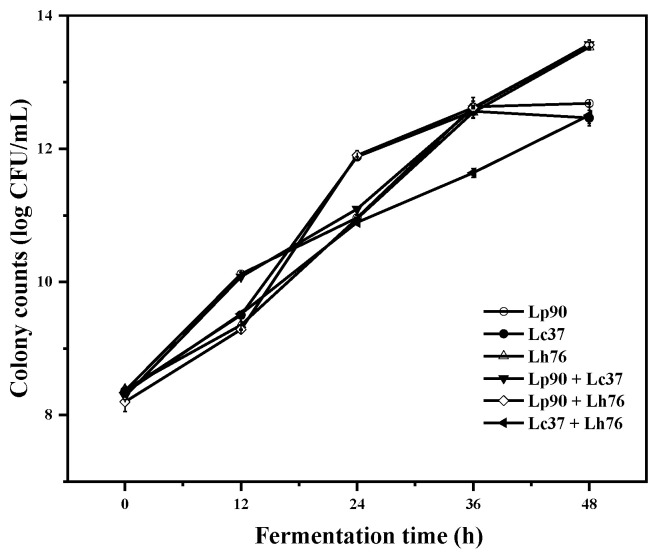
Colony counts of monoculture and binary mixture of lactic acid bacteria in pear juice during fermentation. Abbreviations: Lp90, *Lactiplantibacillus plantarum* 90; Lc37, *Lacticaseibacillus casei* 37; Lh76, *Lactobacillus helveticus* 76; Lp90 + Lc37, mixture of *Lactiplantibacillus plantarum* 90 and *Lacticaseibacillus casei* 37; Lp90 + Lh76, mixture of *Lactiplantibacillus plantarum* 90 and *Lactobacillus helveticus* 76; Lc37 + Lh76, mixture of *Lacticaseibacillus casei* 37 and *Lactobacillus helveticus* 76.

As shown in Figure 1, the proliferation of three *Lactobacillus* strains in monoculture and binary mixture was over five logarithmic cycles, increasing from the initial 8.20 to 12.46–13.56 log CFU/mL. The colony count of monoculture was less than the binary mixture during fermentation because of the symbiosis and/or synergism of different cultures [21]. During the last 12 h of fermentation, the colony count of Lc37 was significantly decreased as a result of the lower pH in pear juice. The colony counts of three *Lactobacillus* strains in monoculture and binary mixture remained far above 10^7^ CFU/mL in the fermented pear juices, suggesting a function as probiotic for health benefits [22].

### 3.2. TSS, pH, Titratable Acid, Organic Acid, and Total Sugar in Pear Juice during Fermentation

Changes in physicochemical parameters of pear juice during LAB fermentation are shown in Figure S1. TSS was decreased sharply for the first 12 h of fermentation, and then slightly decreased until the end of fermentation. Total sugar was decreased significantly at the first 12 h of fermentation from 64.76 g/L to 47.26–57.53 g/L, due to its consumption for growth and metabolism of LAB strains [23]. Then, it showed a slight increase in the last 24 h of fermentation, presumably because of the production of exopolysaccharides by probiotics [7]. Titratable acidity was increased and pH was decreased significantly during fermentation, as a result of the production of organic acid [24].

Malic acid was the major organic acid in unfermented pear juice, while lactic acid was the major organic acid in fermented pear juice (Table 1). Malic acid, oxalic acid, citric acid, and tartaric acid contents were remarkably decreased; meanwhile lactic acid was produced in large amounts after LAB fermentation. Organic acids were important secondary carbon sources for strain proliferation during fermentation [25]. The decrease in titratable acidity for the first 12 h of fermentation should be as a result of the consumption of malic acid, which was converted to lactic acid through malolactic fermentation conducted by LAB strains [9].

### 3.3. Changes in Phytochemical Properties during Fermentation

**Figure 2:**
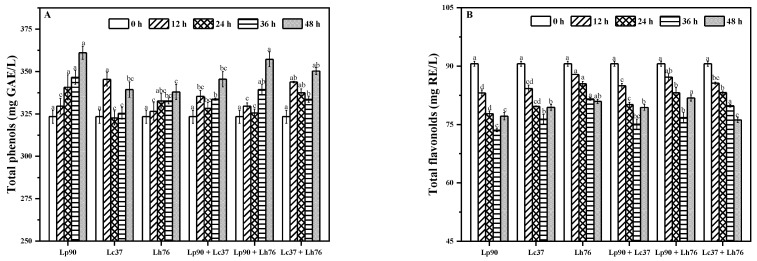
Total phenols (**A**) and flavonoids (**B**) contents in pear juice fermented by monoculture and binary mixture of lactic acid bacteria. Values in the same pattern column with different letters are significantly different (*p* < 0.05). Abbreviations: Lp90, *Lactiplantibacillus plantarum* 90; Lc37, *Lacticaseibacillus casei* 37; Lh76, *Lactobacillus helveticus* 76; Lp90 + Lc37, mixture of *Lactiplantibacillus plantarum* 90 and *Lacticaseibacillus casei* 37; Lp90 + Lh76, mixture of *Lactiplantibacillus plantarum* 90 and *Lactobacillus helveticus* 76; Lc37 + Lh76, mixture of *Lacticaseibacillus casei* 37 and *Lactobacillus helveticus* 76.

As shown in Figure 2, TPC in pear juice was increased whereas TFC was decreased during fermentation. TPC in fermented pear juice ranged from 337.98 (by Lh76) to 361.08 (by Lp90) mg GAE/L, which was higher than unfermented pear juice (323.37 mg GAE/L). The increase in TPC should be attributed to the hydrolysis of large polymeric phenolics into simple new phenolic compounds conducted by LAB strains [1,6,26]. TFC was decreased significantly during fermentation, especially for binary mixture of Lc37 and Lh76 fermentation, decreasing by 15.96%. The changes in TPC and TFC were due to the formation or degradation of phenolic compounds, which should be responsible for antioxidant capacity [27]. It has been proven that LAB strains could produce enzymes to break down cell walls of plant tissue to release bioactive compounds [7]. *L. casei* could produce amylase, lactate dehydrogenases, and proteinase, while *L. plantarum* could produce amylase, β-glucosidase, decarboxylase, lactate dehydrogenases, phenolic acid decarboxylases, phenol reductase, proteinase, and tannase during fermentation [7]. Furthermore, changes of phenolics profile and antioxidant activity of pear juice before and after LAB fermentation were further investigated.

### 3.4. Phenolics Profile and Antioxidant Activity

In this study, 14 phenolic compounds (seven phenolic acids, four flavonoids and three phenolic glycosides) in pear juice were identified (Table 2). Arbutin, vanillic acid, and caffeic acid contents were increased, while syringic acid, p-coumaric acid, chlorogenic acid, ferulic acid, catechin, and epicatechin contents were decreased in pear juice after LAB fermentation. Vanillic acid and caffeic acid appeared, while catechin disappeared in fermented pear juice. Epicatechin and p-coumaric acid were undetectable in pear juice after binary mixture fermentation. LAB strains display an inducible phenolic acid reductase activity, which could hydrogenate the double bond of hydroxycinnamic acids. *L. plantarum* is suggested to promote the biotransformation of protocatechuic acid, caffeic acid, and gallic acid to catechol, dihydro-caffeic acid and pyrogallol, respectively [28].

**Figure 3:**
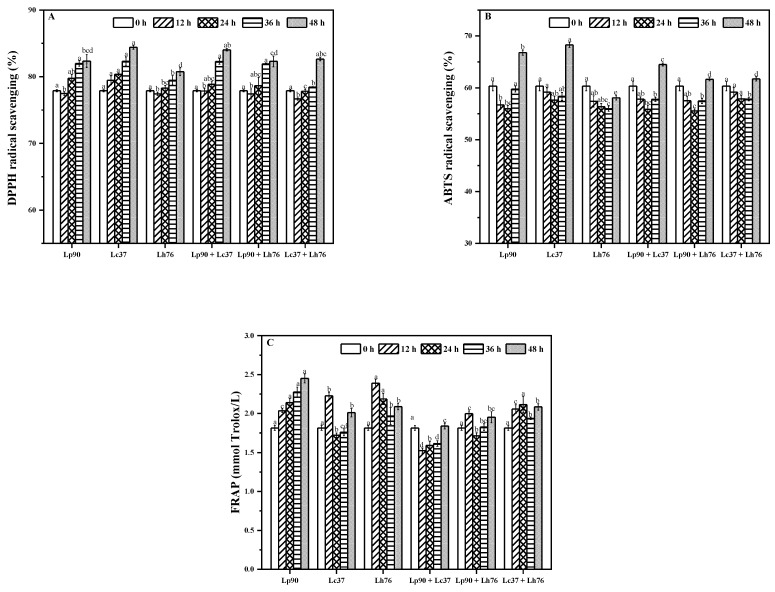
DPPH RSA (**A**), ABTS RSA (**B**) and FRAP (**C**) in pear juice fermented by monoculture and binary mixture of lactic acid bacteria. Values in the same pattern column with different letters are significantly different (*p* < 0.05). Abbreviations: DPPH RSA, 1,1-Diphenyl-2-picrylhydrazyl radical scavenging ability; ABTS RSA, 2, 2’-azino-bis (3-ethylbenzothiazoline-6-sulfonic acid) radical scavenging ability; FRAP, ferric reducing antioxidant power; Lp90, *Lactiplantibacillus plantarum* 90; Lc37, *Lacticaseibacillus casei* 37; Lh76, *Lactobacillus helveticus* 76; Lp90 + Lc37, mixture of *Lactiplantibacillus plantarum* 90 and *Lacticaseibacillus casei* 37; Lp90 + Lh76, mixture of *Lactiplantibacillus plantarum* 90 and *Lactobacillus helveticus* 76; Lc37 + Lh76, mixture of *Lacticaseibacillus casei* 37 and *Lactobacillus helveticus* 76.

**Figure 4:**
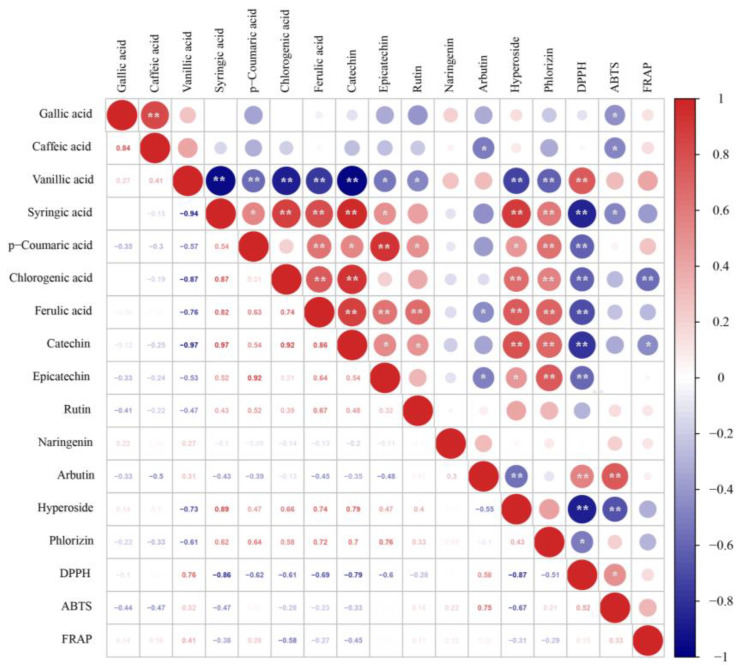
Heat map of Pearson correction coefficient for heat map of phenolics profile and antioxidant activities. * Correlation is significant at *p* < 0.05. ** Correlation is significant at *p* < 0.01.

DPPH, ABTS and FRAP methods are generally used to measure antioxidant capacities of biological materials. As shown in Figure 3, antioxidant activities were increased after fermentation, confirming that LAB strains possessed positive effects on the functional characteristics of pear juice. DPPH RSA slightly decreased in the first 12 h and then increased steadily till the end of fermentation, whereas Lc37 exhibited the highest DPPH RSA and kept increasing throughout fermentation. Significant positive correlations were observed between the phenolic compounds (vanillic acid, R^2^ = 0.76, arbutin, R^2^ = 0.58) and DPPH RSA (Figure 4). ABTS RSA was slightly decreased for the first 36 h and then increased for the last 12 h of fermentation. Significant positive correlation was observed between arbutin content (R^2^ = 0.75) and ABTS RSA. Although FRAP showed fluctuations during LAB fermentation, it was higher in fermented pear juice than unfermented juice.

### 3.5. Flavor Volatiles

In this study, 65 volatiles were detected in pear juice before and after fermentation (Table S1). Alcohols, esters, and aldehydes were the major groups of flavor volatiles in unfermented pear juice, accounting for 20%, 37%, and 29%, respectively. However, alcohols, acids, and esters were the major volatiles in fermented pear juice.

The highest concentration of total volatiles in pear juice was obtained by Lh76 fermentation. It has been reported that *L. helveticus* was an isolate from Emmental cheese, which was consistently associated with improved flavor. Bioinformatic analysis of the genome has confirmed that a plethora of genes act as key metabolic functions for the improvement of cheese flavor [29]. However, Lp90, a single strain with plant isolates, showed the lowest content of total volatile compounds. In addition, binary mixture exhibited higher concentration of volatile compounds compared to monoculture. A recent study has proposed that the use of mixed strains isolated from different sources might improve food flavor [7].

Alcohols were the largest group of volatile compounds identified in all the fermented pear juices, accounting for more than 50% of the total volatile compounds. A total of 15 new alcohols emerged after fermentation. LAB fermentation promoted the production of alcohols, especially for hexanol contributing to the green and apple-skin fragrance. Moreover, (E)-2-hexen-1-ol and linalool were significantly increased, while tert-butanol and 1-nonen-3-ol were decreased after LAB fermentation. (E)-2-hexen-1-ol and linalool endowed fermented pear juice with fresh and fruity notes. Esters play an important role in contributing to fruity notes [30]. In this study, 18 esters were identified which were increased after fermentation by Lh76, binary mixtures of Lp90 and Lh76, and Lc37 and Lh76. The slight increase of esters in FPJ may be associated with the higher availability of the alcohol precursors [31]. The prevailing esters, such as ethyl acetate, ethyl butyrate, ethyl 2-methyl butyrate, (E)-ethyl tiglate were characterized by positive fruity notes. Aldehydes and ketones were decreased in all the fermented pear juices, giving positive aroma attributes to the flavor of juices. They were suggested to have been reduced or oxidized to alcohols or acids by LAB metabolism [25]. It has been proven that acetaldehyde contributes fruity notes at lower concentration, while negatively affecting the aroma of juice when above 200 mg/L [31]. Acetic acid was the main component of the volatile acids that provided sourness, which was only detected in fermented pear juices, except Lp90. A significant increase in terpenoids in fermented pear juice was observed, and three new terpenoids including (E)-β-ocimene, β-ocimene and γ-terpinene were produced by Lp90 and Lc37 fermentations. Terpenoids, with pine and citrus notes, exhibit a positive correlation with customer preference [31].

**Figure 5:**
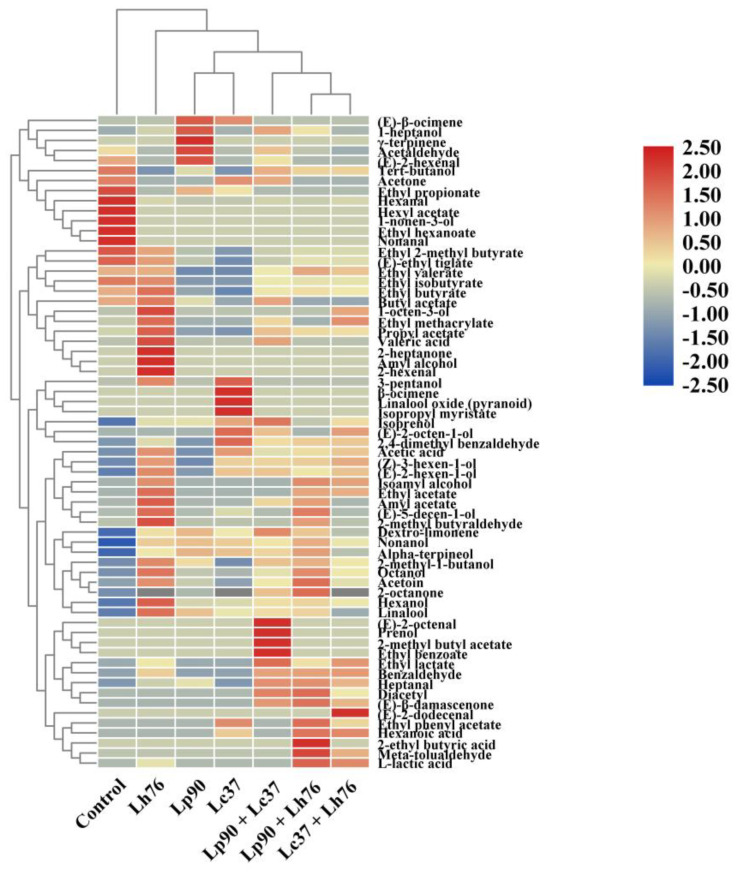
Hierarchical cluster and heat map of volatiles profile in unfermented and fermented pear juices by monoculture and binary mixture of lactic acid bacteria. The color scale represents the scaled abundance of each variable, denoted as d^2^ (squared Euclidean distance), with red indicating high abundance and blue indicating low abundance. Abbreviations: Lp90, *Lactiplantibacillus plantarum* 90; Lc37, *Lacticaseibacillus casei* 37; Lh76, *Lactobacillus helveticus* 76; Lp90 + Lc37, mixture of *Lactiplantibacillus plantarum* 90 and *Lacticaseibacillus casei* 37; Lp90 + Lh76, mixture of *Lactiplantibacillus plantarum* 90 and *Lactobacillus helveticus* 76; Lc37 + Lh76, mixture of *Lacticaseibacillus casei* 37 and *Lactobacillus helveticus* 76.

The volatile compounds profile of each sample was evaluated by hierarchical clustering analysis and drove the clustering according to Euclidean distance (Figure 5). Pear juices were clustered based on the specific metabolic profile of LAB strains and grouped into four clusters. The first cluster was unfermented pear juice, which had the lowest volatile volatiles. The second cluster was pear juice fermented by Lh76, which possessed a wide range of volatile compounds. The remaining two clusters, single strain (Lp90 and Lc37) and binary mixtures, exhibited different aroma compositions. The results suggested that lactic acid fermentation by Lh76 or binary mixtures could provide a rich and pleasant aroma, increasing the consumer acceptability of fermented pear juice.

### 3.6. Color Properties

Color is a crucial characteristic, because it provides much intuitive information about the quality of fruit juice [20]. As shown in Table S2, L* value was decreased in all the fermented pear juices, indicating a “dark” luminosity, while a* and b* values were increased which suggesting a turning to brown after fermentation. Fruit browning is attributed to quinine formation as a result of phenolic oxidation by polyphenol oxidase [32]. Significant differences of Δ*E* (*p* < 0.05) were observed, and the smallest value (5.09) was found by Lc37 fermentation, suggesting the color closest to unfermented pear juice. It has been reported that changes in color parameters were related to TPC and TFC [12]. Therefore, correlations were investigated among the phytochemical contents and color parameters (Table S3). Results exhibited that TFC was significantly and positively correlated with L* value (*p* < 0.01), while negatively correlated with a* and b* values. TPC was significantly and negatively correlated with L* value, while positively correlated with b* value.

### 3.7. Sensory Evaluation

As shown in Table S4, significant differences were observed in color, aroma, sweetness, sourness, and overall acceptability among pear juices (*p* < 0.05). Unfermented pear juice and fermented juice were acceptable with scores ranging from 6.12 to 8.48 which were higher than the acceptability limit (6.0) [20].

The scores of overall acceptability obtained from a 15-member semi-trained panel were listed in the following order: pear juices fermented by binary mixture > pear juices fermented by monoculture fermentation > unfermented pear juice. In sensory evaluation, the slight differences in color were due to the browning of pear juices, but nevertheless were still well accepted by panelists. The unfermented pear juice showed the highest sweetness and the lowest sourness among samples, relating to sugars and organic acids, respectively. LAB fermentation can convert sugars (mainly glucose and fructose) to organic acids and aroma constituents, contributing to the flavor formation of fermented juices.

## 4. Conclusions

The present study provides an approach to preparing functional pear juice by monoculture and binary mixture of three probiotics (Lp90, Lh76, and Lc37) fermentation. Pear juice was an excellent matrix for probiotics and the colony counts in the fermented juice reached 12.46–13.56 log CFU/mL, a mean of bacteria load which can provide health benefits. Binary mixture exhibited better proliferation ability than monoculture in pear juice. Antioxidant activities (DPPH RSA, ABTS RSA, and FRAP) were dramatically improved by LAB fermentation, especially for binary mixture of Lp90 and Lc37 fermentation, as a result of the improvement in vanillic acid and arbutin contents in fermented pear juices. Furthermore, flavor volatiles and aroma complexity were improved by LAB fermentation, due to the increase in contents of alcohols, esters, acids and terpenoids, decrease in contents of aldehydes and ketones, and the formation of 30 new volatile compounds in the fermented pear juice. Further study should investigate the viability of monoculture and binary mixture, phenolics profile, and antioxidant activity in fermented pear juice using simulated gastrointestinal digestibility.

## Figures and Tables

**Table 1:** Organic acids in pear juice fermented by monoculture and binary mixture of lactic acid bacteria (mg/mL).

Organic Acids	RT (min)	Control	Lp90	Lc37	Lh76	Lp90 + Lc37	Lp90 + Lh76	Lc37 + Lh76
Oxalic acid	4.89	0.27 ± 0.00 ^a^	0.25 ± 0.01 ^a,b^	0.23 ± 0.01 ^b^	0.24 ± 0.02 ^b^	0.24 ± 0.01 ^b^	0.23 ± 0.00 ^b^	0.24 ± 0.01 ^b^
Tartaric acid	5.34	0.54 ± 0.02 ^a^	0.28 ± 0.02 ^b^	0.23 ± 0.02 ^c,d^	0.20 ± 0.01 ^c,d^	0.25 ± 0.02 ^b,c^	0.18 ± 0.01 ^d^	0.23 ± 0.02 ^b,c^
Malic acid	6.83	2.09 ± 0.09 ^a^	ND	ND	ND	ND	ND	ND
Lactic acid	8.36	ND	7.79 ± 0.07 ^a^	5.83 ± 0.03 ^e^	7.02 ± 0.01 ^c^	7.41 ± 0.04 ^b^	6.64 ± 0.05 ^d^	7.39 ± 0.19 ^b^
Citric acid	10.59	0.26 ± 0.01 ^a^	0.13 ± 0.01 ^b^	0.04 ± 0.01 ^e^	0.10 ± 0.00 ^b^	0.06 ± 0.00 ^d^	0.03 ± 0.00 ^e^	0.07 ± 0.00 ^d^

Values in the same row with different letters are significantly different (*p* < 0.05). ND, not detectable. Abbreviations: Lp90, *Lactiplantibacillus plantarum* 90; Lc37, *Lacticaseibacillus casei* 37; Lh76, *Lactobacillus helveticus* 76; Lp90 + Lc37, mixture of *Lactiplantibacillus plantarum* 90 and *Lacticaseibacillus casei* 37; Lp90 + Lh76, mixture of *Lactiplantibacillus plantarum* 90 and *Lactobacillus helveticus* 76; Lc37 + Lh76, mixture of *Lacticaseibacillus casei* 37 and *Lactobacillus helveticus* 76.

**Table 2:** Phenolics profile in pear juice fermented by monoculture and binary mixture of lactic acid bacteria (mg/L).

	RT (min)	Control	Lp90	Lc37	Lh76	Lp90 + Lc37	Lp90 + Lh76	Lc37 + Lh76
*Phenolic acids*								
Gallic acid	3.15	22.40 ± 0.22 ^c,d^	21.93 ± 0.46 ^d^	21.74 ± 0.19 ^d,e^	23.35 ± 0.05 ^b,c^	20.66 ± 0.65 ^e^	23.74 ± 0.73 ^b^	26.82 ± 0.36 ^a^
Caffeic acid	4.76	ND	ND	ND	3.22 ± 0.03 ^b^	ND	ND	5.55 ± 0.31 ^a^
Vanillic acid	22.19	ND	0.67 ± 0.05 ^c^	0.77 ± 0.01 ^a,b^	0.72 ± 0.02 ^b,c^	0.71 ± 0.03 ^b,c^	0.66 ± 0.05 ^c^	0.85 ± 0.05 ^a^
Syringic acid	24.64	2.30 ± 0.13 ^a^	0.98 ± 0.01 ^b,c^	0.81 ± 0.00 ^c^	1.12 ± 0.03 ^b^	0.85 ± 0.05 ^c^	1.06 ± 0.12 ^b^	0.98 ± 0.09 ^b,c^
p-Coumaric acid	35.21	0.49 ± 0.02 ^a^	0.42 ± 0.03 ^b^	0.31 ± 0.02 ^c^	0.33 ± 0.01 ^c^	ND	ND	ND
Chlorogenic acid	36.27	26.04 ± 0.58 ^a^	2.74 ± 0.04 ^e^	3.66 ± 0.06 ^d^	ND	8.21 ± 0.27 ^b,c^	7.65 ± 0.16 ^c^	8.87 ± 0.25 ^b^
Ferulic acid	37.68	0.99 ± 0.07 ^a^	0.34 ± 0.01 ^c^	0.38 ± 0.01 ^b,c^	0.40 ± 0.00 ^b,c^	0.33 ± 0.02 ^c^	ND	0.44 ± 0.01 ^b^
*Flavonoids*								
Catechin	15.40	256.99 ± 0.73 ^a^	ND	ND	ND	ND	ND	ND
Epicatechin	28.14	4.77 ± 0.02 ^a^	2.50 ± 0.09 ^d^	4.16 ± 0.04 ^b^	3.71 ± 0.12 ^c^	ND	ND	ND
Rutin	37.87	10.88 ± 0.25 ^a^	10.81 ± 0.05 ^a^	9.51 ± 0.03 ^b^	9.72 ± 0.13 ^b^	10.59 ± 0.18 ^a^	8.69 ± 0.28 ^c^	9.69 ± 0.21 ^b^
Naringenin	51.56	1.90 ± 0.14 ^a^	1.94 ± 0.13 ^a^	1.95 ± 0.05 ^a^	1.92 ± 0.04 ^a^	1.93 ± 0.07 ^a^	1.95 ± 0.06 ^a^	1.95 ± 0.05 ^a^
*Phenolic glycoside*								
Arbutin	2.63	215.74 ± 0.76 ^c,d^	225.43 ± 0.56 ^ab^	224.48 ± 0.46 ^a,b^	210.26 ± 1.30 ^d^	228.91 ± 2.42 ^a^	224.82 ± 4.66 ^a,b^	219.06 ± 4.04 ^b,c^
Hyperoside	38.52	2.83 ± 0.04 ^a^	2.19 ± 0.10 ^c^	2.08 ± 0.08 ^c^	2.53 ± 0.02 ^a,b^	2.21 ± 0.07 ^c^	2.23 ± 0.18 ^b,c^	2.29 ± 0.17 ^b,c^
Phlorizin	43.80	0.32 ± 0.01 ^a^	0.22 ± 0.00 ^b^	0.30 ± 0.00 ^a^	0.21 ± 0.02 ^b^	0.21 ± 0.02 ^b^	0.20 ± 0.02 ^b^	0.21 ± 0.01 ^b^

Values in the same row with different letters are significantly different (*p* < 0.05). ND, not detectable. Abbreviations: Lp90, *Lactiplantibacillus plantarum* 90; Lc37, *Lacticaseibacillus casei* 37; Lh76, *Lactobacillus helveticus* 76; Lp90 + Lc37, mixture of *Lactiplantibacillus plantarum* 90 and *Lacticaseibacillus casei* 37; Lp90 + Lh76, mixture of *Lactiplantibacillus plantarum* 90 and *Lactobacillus helveticus* 76; Lc37 + Lh76, mixture of *Lacticaseibacillus casei* 37 and *Lactobacillus helveticus* 76.

## Data Availability

Not applicable.

## Supplementary Materials

The following are available online at https://www.mdpi.com/article/10.3390/foods11010011/s1, Figure S1: Changes in soluble solids (A), total sugar (B), titratable acidity (C) and pH value (D) during pear juice fermentation by monoculture and binary mixture of lactic acid bacteria; Table S1: Flavor volatiles in pear juice fermented by monoculture and binary mixture of lactic acid bacteria; Table S2: Colorimetric properties of pear juice fermented by monoculture and binary mixture of lactic acid bacteria; Table S3: Pearson′s correlation coefficients of TPC, TFC and colorimetric properties; Table S4: Sensory evaluation of pear juice fermented by monoculture and binary mixture of lactic acid bacteria.
