# Supplementary material for: Phenolics Profile, Antioxidant Activity and Flavor Volatiles of Pear Juice: Influence of Lactic Acid Fermentation Using Three Lactobacillus Strains in Monoculture and Binary Mixture
Source: Foods. 2021 Dec 21;11(1):11. doi: 10.3390/foods11010011 (PMC8750113; doi:10.3390/foods11010011)
Supplement: Supplementary file 1 [file foods-11-00011-s001.zip › foods-1513394-supplementary.pdf]

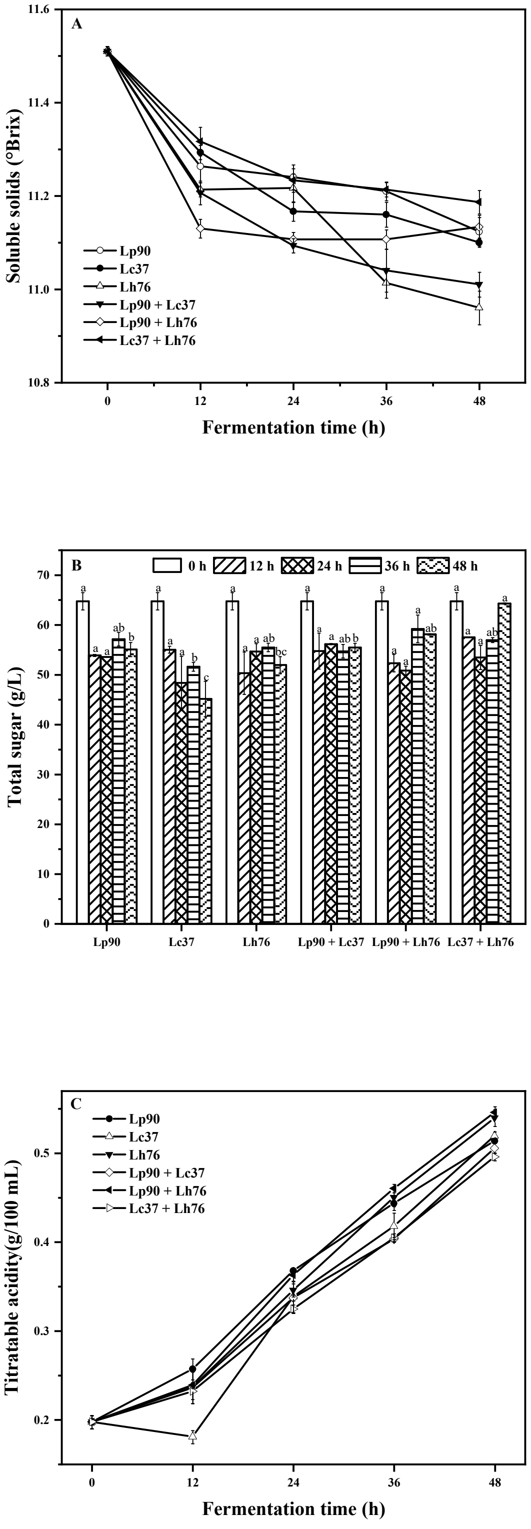
A
Soluble solids (°Brix)
Fermentation time (h)
Lp90
Lc37
Lh76
Lp90 + Lc37
Lp90 + Lh76
Lc37 + Lh76
B
0 h
12 h
24 h
36 h
48 h
Total sugar (g/L)
Lp90
Lc37
Lh76
Lp90 + Lc37
Lp90 + Lh76
Lc37 + Lh76
C
Titratable acidity(g/100 mL)
Fermentation time (h)
Lp90
Lc37
Lh76
Lp90 + Lc37
Lp90 + Lh76
Lc37 + Lh76

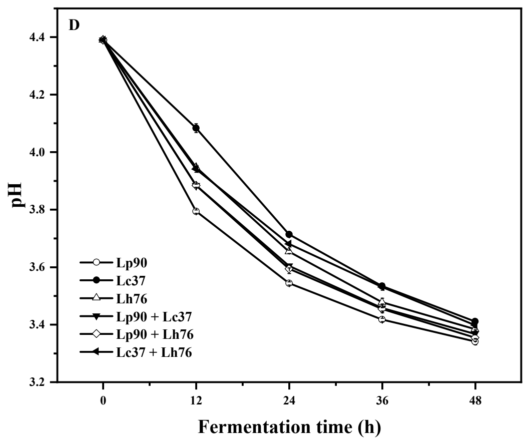

Figure S1. Changes in soluble solids (A), total sugar (B), titratable acidity (C) and pH value (D) during pear juice fermentation by monoculture and binary mixture of lactic acid bacteria. Abbreviations: Lp90, *Lactiplantibacillus plantarum* 90; Lc37, *Lacticaseibacillus casei* 37; Lh76, *Lactobacillus helveticus* 76; Lp90 + Lc37, mixture of *Lactiplantibacillus plantarum* 90 and *Lacticaseibacillus casei* 37; Lp90 + Lh76, mixture of *Lactiplantibacillus plantarum* 90 and *Lactobacillus helveticus* 76; Lc37 + Lh76, mixture of *Lacticaseibacillus casei* 37 and *Lactobacillus helveticus* 76.

Table S1. Flavor volatiles in pear juice fermented by monoculture and binary mixture of lactic acid bacteria (μg/L)

	Flavor volatiles	RT (min)	Control	Lp90	Lc37	Lh76	Lp90 + Lc37	Lp90 + Lh76	Lc37 + Lh76	Odor description
Alcohols										
1	Tert-butanol	5.35	356.94	145.31	ND	ND	271.31	212.97	216.63	camphor
2	3-pentanol	5.37	ND	ND	246.96	196.21	ND	ND	ND	sweet herbal oily nutty
3	Isoprenol	9.62	ND	46.42	73.51	45.27	89.68	35.52	55.56	sweet fruity
4	Isoamyl alcohol	9.80	ND	ND	ND	45.76	ND	46.42	40.00	fermented, banana
5	2-methyl-1-butanol	9.92	ND	51.03	ND	83.47	61.49	68.53	47.43	wine, onion
6	Amyl alcohol	10.97	ND	ND	ND	58.97	ND	ND	ND	fermented, bready, cereal
7	Prenol	11.30	ND	ND	ND	ND	23.31	ND	ND	green, fruity
8	(Z)-3-hexen-1-ol	14.59	ND	ND	105.17	142.55	101.96	107.03	130.22	fresh, green, raw fruity
9	(E)-2-hexen-1-ol	15.07	159.14	549.04	2177.36	2871.24	2175.04	1808.35	2213.60	green leafy, fresh, fatty
10	Hexanol	15.19	2219.55	6205.40	6364.16	11814.31	7511.94	7856.32	7017.75	green, apple-skin, oily
11	1-heptanol	19.74	ND	2696.24	64.34	565.65	1783.14	1036.33	145.25	herbal, green, peony
12	1-octen-3-ol	20.20	ND	ND	ND	73.87	ND	ND	41.17	mushroom, earthy, green
13	(E)-2-octen-1-ol	24.05	ND	ND	49.22	ND	27.80	ND	36.30	green, citrus, vegetable
14	Octanol	24.17	ND	129.04	86.10	429.63	189.08	388.27	215.02	waxy, green, citrus
15	Linalool	25.44	408.86	1010.33	852.55	1284.09	918.91	947.68	608.55	citrus, orange, lemon
16	Nonanol	28.06	ND	419.85	396.91	394.48	349.02	453.93	329.91	waxy, citrus, rue
17	Alpha-terpineol	28.83	ND	88.54	82.78	68.02	75.09	96.54	45.92	lemon, lime, soapy

18	1-nonen-3-ol	29.21	294.56	ND	ND	ND	ND	ND	ND	earthy, mushroom, green
19	(E)-5-decen-1-ol	30.91	ND	ND	46.56	228.52	ND	206.74	ND	waxy, floral, rose
20	Linalool oxide (pyranoid)	33.60	ND	ND	227.79	ND	ND	ND	ND	floral, honey
	Subtotal		3439.06	11341.20	10773.42	18302.06	13577.75	13264.63	11143.32	
Esters										
1	Ethyl acetate	6.54	ND	ND	ND	5467.88	ND	3881.84	3593.79	grape, cherry nuance
2	Ethyl propionate	8.97	139.94	74.19	43.99	ND	ND	ND	ND	winey, apple, grape
3	Propyl acetate	9.04	79.38	52.85	45.58	151.08	109.76	99.66	97.06	tutti-frutti, banana, honey
4	Ethyl isobutyrate	10.70	91.07	ND	ND	84.17	42.35	38.53	39.85	pungent, fruity
5	Ethyl methacrylate	11.66	28.11	ND	ND	176.40	83.43	ND	144.49	acrylate
6	Ethyl butyrate	12.24	2372.43	926.91	528.06	2983.82	1744.12	1906.73	1810.91	tutti-frutti, apple, fresh
7	Butyl acetate	12.78	65.66	29.82	ND	86.87	68.20	ND	ND	sweet, banana, tutti-frutti,
8	Ethyl lactate	12.66	ND	ND	ND	16.40	39.84	17.51	31.65	sweet, fruity, creamy
9	Ethyl 2-methyl butyrate	14.44	655.12	217.40	104.74	487.67	254.27	283.51	284.10	berry, pineapple, mango
10	2-methyl butyl acetate	15.64	ND	ND	ND	ND	10.36	ND	ND	sweet, banana, fruity
11	Ethyl valerate	16.57	87.57	ND	ND	89.18	58.88	92.24	78.73	strawberry, sweet, pineapple
12	Amyl acetate	17.13	ND	ND	ND	24.75	10.25	16.86	ND	sweet, pear, banana
13	(E)-ethyl tiglate	18.24	659.14	352.73	234.01	559.24	363.39	410.81	402.46	sweet, green, tutti-frutti
14	Ethyl hexanoate	21.00	1287.25	ND	ND	ND	ND	ND	ND	sweet, pineapple, fruity
15	Hexyl acetate	21.59	1013.32	ND	ND	ND	ND	ND	ND	fruity, green, sweet, pear

16	Ethyl benzoate	27.82	ND	ND	ND	ND	19.12	ND	ND	sweet, minty, birch, beer
17	Ethyl phenyl acetate	30.16	ND	ND	34.17	ND	ND	40.46	18.68	rosy honey, balsamic cocoa
18	Isopropyl myristate	44.49	ND	ND	22.37	ND	ND	ND	ND	oily fatty
	Subtotal		6479.00	1653.90	1012.92	10127.45	2803.98	6788.14	6501.72	
Aldehydes										
1	Acetaldehyde	4.55	93.57	145.13	68.70	67.20	103.17	71.81	61.41	pungent, fresh
2	2-methyl butyraldehyde	7.55	ND	ND	ND	26.02	ND	16.20	ND	musty, nutty, cereal, caramel
3	Hexanal	11.94	4082.15	112.89	129.20	444.84	375.58	240.51	436.30	woody, apple, grassy, citrus
4	(E)-2-hexenal	14.03	673.08	1123.36	ND	ND	376.08	ND	ND	fresh green, leafy, fruity
5	2-hexenal	14.03	ND	ND	ND	416.43	ND	ND	ND	sweet, almond, fruity, green
6	Heptanal	16.38	ND	93.24	ND	71.35	191.40	193.84	155.66	green, oily, cortex, grassy
7	Benzaldehyde	18.71	ND	ND	ND	170.90	230.58	232.47	243.48	sweet, cherry, nutty, woody
8	(E)-2-octenal	23.31	ND	ND	ND	ND	25.40	ND	ND	sweet, green, citrus peel, spicy
9	(E)-2-dodecenal	23.31	ND	ND	ND	ND	ND	ND	29.18	waxy, citrus peel, bitter
10	Meta-tolualdehyde	23.65	ND	ND	ND	ND	ND	55.69	27.90	fruity, cherry, bitter almond
11	Nonanal	25.38	314.28	ND	ND	ND	ND	ND	ND	citrus, cucumber, melon
12	2,4-dimethyl benzaldehyde	29.30	ND	ND	224.28	83.04	113.38	130.65	134.27	cherry, almond, spice, vanilla
	Subtotal		5163.08	1474.62	422.19	1279.78	1415.58	941.17	1088.18	
Ketones										
1	Acetone	5.02	2243.81	ND	2042.70	ND	1724.08	ND	ND	ethereal, apple, pear

2	Diacetyl	6.00	ND	ND	ND	ND	416.76	474.88	153.83	sweet, buttery, creamy, milky
3	Acetoin	8.61	ND	62.61	ND	202.45	106.64	240.33	88.51	creamy, dairy, sweet, milky
4	2-heptanone	15.92	ND	ND	ND	157.49	ND	ND	ND	cheese, fruity, coconut, waxy
5	2-octanone	20.49	13.60	25.50			49.11	67.87		dairy, waxy, cheese, woody
6	(E)-β-damascenone	34.69	ND	ND	ND	ND	72.82	91.30	58.37	apple, rose, honey, tobacco
	Subtotal		2257.41	88.11	2042.70	359.94	2369.41	874.37	300.72	
Acids										
1	Acetic acid	6.84	ND	ND	3689.14	3842.83	1873.18	2438.93	2831.43	pungent, sour, overripe fruit
2	2-ethyl butyric acid	13.76	ND	ND	ND	ND	ND	94.47	ND	acidic, tropical fruity, creamy
3	L-lactic acid	15.79	ND	ND	ND	229.68	ND	903.13	727.33	sour
4	Valeric acid	20.11	ND	ND	ND	121.43	71.57	ND	ND	dairy-like with milky, cheese
5	Hexanoic acid	20.16	ND	ND	52.53	ND	ND	98.40	87.90	cheesy, fruity, fatty
	Subtotal		0.00	0.00	3741.67	4193.94	1944.75	3534.93	3646.66	
Terpenoids										
1	Dextro-limonene	22.87	166.16	270.66	244.76	250.71	292.30	262.73	219.66	sweet, orange, citrus
2	(E)-β-ocimene	23.02	ND	48.93	36.69	ND	ND	ND	ND	sweet, herbal
3	β-ocimene	23.52	ND	ND	80.17	ND	ND	ND	ND	green, tropical, woody, floral
4	γ-terpinene	24.06	ND	17.98	ND	ND	ND	ND	ND	citrus, lime-like, oily
	Subtotal		166.16	337.57	361.62	250.71	292.30	262.73	219.66	
	Total		17504.71	14895.40	18354.51	34513.88	22403.78	25665.97	22900.27	

Results are the mean value of two replicates. Standard errors were always lower than 10% of mean value. Odor descriptions were cited from www.flavornet.org. ND, not detectable. Abbreviations: Lp90, *Lactiplantibacillus plantarum* 90; Lc37, *Lacticaseibacillus casei* 37; Lh76, *Lactobacillus helveticus* 76; Lp90 + Lc37, mixture of *Lactiplantibacillus plantarum* 90 and *Lacticaseibacillus casei* 37; Lp90 + Lh76, mixture of *Lactiplantibacillus plantarum* 90 and *Lactobacillus helveticus* 76; Lc37 + Lh76, mixture of *Lacticaseibacillus casei* 37 and *Lactobacillus helveticus* 76.

Table S2. Colorimetric properties of pear juice fermented by monoculture and binary mixture of lactic acid bacteria

	L^*	a^*	b^*	ΔE
Control	94.58 ± 0.03[a]	-0.79 ± 0.01[g]	10.88 ± 0.01[g]	-
Lp90	91.23 ± 0.02[c]	0.27 ± 0.01[e]	18.21 ± 0.01[d]	8.13 ± 0.01[e]
Lc37	92.48 ± 0.04[b]	0.10 ± 0.01[f]	15.43 ± 0.01[f]	5.09 ± 0.02[g]
Lh76	88.82 ± 0.04[h]	1.89 ± 0.02[a]	18.91 ± 0.03[b]	10.24 ± 0.05[b]
Lp90 + Lc37	90.93 ± 0.02[d]	0.50 ± 0.01[d]	16.93 ± 0.02[e]	7.19 ± 0.01[f]
Lp90 + Lh76	89.62 ± 0.04[g]	1.37 ± 0.01[c]	18.92 ± 0.01[b]	9.70 ± 0.03[c]
Lc37 + Lh76	89.72 ± 0.02[f]	1.43 ± 0.00[b]	18.67 ± 0.01[c]	9.45 ± 0.01[d]

L^*-Lightness, a^*-Redness, b^*-Yellowness, ΔE-color difference.

Values in the same column with different letters are significantly different ($p < 0.05$). Abbreviations: Lp90, *Lactiplantibacillus plantarum* 90; Lc37, *Lacticaseibacillus casei* 37; Lh76, *Lactobacillus helveticus* 76; Lp90 + Lc37, mixture of *Lactiplantibacillus plantarum* 90 and *Lacticaseibacillus casei* 37; Lp90 + Lh76, mixture of *Lactiplantibacillus plantarum* 90 and *Lactobacillus helveticus* 76; Lc37 + Lh76, mixture of *Lacticaseibacillus casei* 37 and *Lactobacillus helveticus* 76.

Table S3. Pearson's correlation coefficients of TPC, TFC and colorimetric properties

	**TPC**	**TFC**	***L****	***a****	***b****
TPC	1				
TFC	-0.746**	1			
*L**	-0.596**	0.661**	1		
*a**	0.360	-0.464*	-0.903**	1	
*b**	0.783**	-0.813**	-0.927**	0.704**	1

* Correlation is significant at $p < 0.05$. ** Correlation is significant at $p < 0.01$. Abbreviations: TPC, total phenolic content; TFC, total flavonoid content.

Table S4. Sensory evaluation of pear juice fermented by monoculture and binary mixture of lactic acid bacteria

Characteristic	Control	Lp90	Lc37	Lh76	Lp90 + Lc37	Lp90 + Lh76	Lc37 + Lh76
Color	8.12 ± 0.17^{a}	7.53 ± 0.29^{b}	8.05 ± 0.16^{a}	7.15 ± 0.28^{c}	7.94 ± 0.28^{a}	7.65 ± 0.27^{b}	7.61 ± 0.29^{b}
Aroma	6.21 ± 0.15^{c}	6.87 ± 0.17^{d}	7.02 ± 0.13^{d}	8.31 ± 0.16^{a}	7.85 ± 0.19^{b}	7.83 ± 0.16^{b}	7.63 ± 0.19^{c}
Sweetness	7.56 ± 0.11^{a}	6.95 ± 0.12^{d}	7.01 ± 0.12^{cd}	6.74 ± 0.16^{e}	6.96 ± 0.15^{d}	7.15 ± 0.12^{b}	7.12 ± 0.11^{bc}
Sourness	6.12 ± 0.15^{d}	7.42 ± 0.14^{ab}	7.23 ± 0.22^{c}	7.53 ± 0.17^{ab}	7.52 ± 0.17^{ab}	7.37 ± 0.13^{bc}	7.57 ± 0.15^{a}
Acceptability	7.05 ± 0.20^{a}	7.61 ± 0.22^{b}	7.71 ± 0.23^{b}	8.03 ± 0.14^{b}	8.32 ± 0.15^{c}	8.31 ± 0.15^{c}	8.48 ± 0.13^{c}

Values in the same row with different superscript letters are significantly different ($p < 0.05$). Abbreviations: Lp90, *Lactiplantibacillus plantarum* 90; Lc37, *Lacticaseibacillus casei* 37; Lh76, *Lactobacillus helveticus* 76; Lp90 + Lc37, mixture of *Lactiplantibacillus plantarum* 90 and *Lacticaseibacillus casei* 37; Lp90 + Lh76, mixture of *Lactiplantibacillus plantarum* 90 and *Lactobacillus helveticus* 76; Lc37 + Lh76, mixture of *Lacticaseibacillus casei* 37 and *Lactobacillus helveticus* 76.
